# Toxicity Risk Assessment Due to Particulate Matter Pollution from Regional Health Data: Case Study from Central Romania

**DOI:** 10.3390/toxics12020137

**Published:** 2024-02-07

**Authors:** Carmen Maftei, Ashok Vaseashta, Ionut Poinareanu

**Affiliations:** 1 Faculty of Civil Engineering, Transilvania University of Brasov, 900152 Brasov, Romania; 2 Office of Research, International Clean Water Institute, Manassas, VA 20108, USA; 3 Institute of Biomedical Engineering and Nanotechnologies, Faculty of Mechanical Engineering, Transport and Aeronautics, Ķīpsalas, LV1048 Rīga, Latvia; 4 Faculty of Medicine, “Ovidius” University of Constanta, 900470 Constanta, Romania; 5 Clinical Service of Pathology, “St. Apostol Andrei” Emergency County Hospital, 145 Tomis Blvd., 900591 Constanta, Romania; 6 Faculty of Materials Science and Engineering, Transilvania University of Brasov, 500036 Brasov, Romania

**Keywords:** PM, risk assessment, central Romania, cardiovascular, health, indicators

## Abstract

Air pollution poses one of the greatest dangers to public well-being. This article outlines a study conducted in the Central Romania Region regarding the health risks associated with particulate matter (PM) of two sizes, viz., PM_10_ and PM_2.5_. The methodology used consists of the following: (i) an analysis of the effects of PM pollutants, (ii) an analysis of total mortality and cardiovascular-related mortality, and (iii) a general health risk assessment. The Central Region of Romania is situated in the Carpathian Mountains’ inner arch (consisting of six counties). The total population of the region under investigation is about 2.6 million inhabitants. Health risk assessment is calculated based on the relative risk (RR) formula. During the study period, our simulations show that reducing these pollutants’ concentrations below the new WHO guidelines (2021) will prevent over 172 total fatalities in Brasov alone, as an example. Furthermore, the potential benefit of reducing annual PM_2.5_ levels on total cardiovascular mortality is around 188 persons in Brasov. Although health benefits may also depend upon other physiological parameters, all general health indicators point towards a significant improvement in overall health by a general reduction in particulate matter, as is shown by the toxicity assessment of the particulate matter in the region of interest. The modality can be applied to other locations for similar studies.

## 1. Introduction

Particulate matter (PM) is classified according to its diameters, viz., that with a diameter of 10 microns or less (PM_10_), while fine particulate matter is defined as particles that are 2.5 microns or less in diameter (PM_2.5_); thus, PM_2.5_ comprises a portion of PM_10_. The common sources of PM_10_ include manufacturing industries, construction, and fossil fuel combustion, such as diesel exhaust particles (DEP) and emissions from coal-burning stoves [1]. PM_10_ is inhalable into the lungs and can induce adverse health effects, while exposure to fine PM_2.5_ aggravates cardiovascular disease (CVD), among other physiological effects. A recent study conducted by the authors [2] demonstrates that the key sectors responsible for polluting the air in Brasov with PM_10_ and PM_2.5_ are commercial, institutional, and households (61.2% and 48.2%, respectively), manufacturing industries and construction (14.2% and 11.1%, respectively), transportation (11.8% and 10.6%, respectively), mineral products (12.3% and 29.6%, respectively), and energy production and distribution (~0.3%). In general, airborne PM varies widely in size, shape, and chemical composition. Particles are defined by their diameter for air quality regulatory purposes since a wide range of adverse health effects have been linked to air pollution exposure. A publication by the World Health Organization (WHO) [3] asserts that many health effects are associated with air pollution, such as mortality caused by chronic cardiovascular and respiratory diseases, chronic obstructive pulmonary disease (COPD), etc. A study reported by the Global Burden of Diseases (GBD) reveals that the global level of total death for the 1990–2019 period represented by cardiovascular disease (CVD) (GBD Compare, 2022) was at 32.84%, while, for the same period, at the Romanian level, the percent was 57.26%. Further studies conducted around the globe [4,5,6] show that air pollution, and especially exposure to PM_2.5_, exacerbates CVD, resulting in a high mortality rate.

Several factors that generally contribute to CVD are certain lifestyles, such as obesity, alcohol consumption, and the use of tobacco [7], but several recent epidemiological studies show that air pollution could also contribute to an increased risk of CVD. In 2006, Pope et al. [8] showed that short-term exposure to PM_2.5_ could be associated with acute ischemic heart disease (IHD) events. The research literature also suggests that increased PM concentrations are linked to higher rates of morbidity and mortality among EU countries. According to Orru [9], in Estonia, PM represents a public health concern, leading to an annual increase in estimated premature deaths, a decrease in life expectancy, and an increase in the number of hospitalizations. Exposure to PM_2.5_ is a major cause of premature deaths worldwide, as compared to previous estimations of mortality due to this pollutant and is significantly higher (~50%) according to studies by Anenberg et al. [10]. The Aphekom project concludes that EU citizens are continuously exposed to air pollution of PM_2.5_ exceeding the WHO limits, and it was concluded that life expectancy at age 30 could be reduced by 22 months [11].

Airborne particulate matter consists of a mixture of many chemical species, viz., solids, aerosols of small droplets of liquid, dry solid fragments, and solid cores with liquid coatings, particles of varying sizes, shapes, and chemical compositions, and may contain inorganic ions, metallic compounds, elemental carbon, organic compounds, and compounds from the earth’s crust. The relative risk (*RR*) is a widely used function to estimate the health impact of different pollutants. Different studies suggest the logarithmic model recommended by WHO [8,10,12,13,14,15,16]. Concerning the relative risk (*RR*) for the long-term impact of PM_2.5_, Pope [15] recommends a value of 1.06 (95% CI—confidence interval, which represents a range of estimates for an unknown parameter in frequentist statistics—1.02–1.11) per 10 μg/m^3^ (total non-external causes mortality) and a value of 1.12 (95% CI 1.08–1.15) per 10 μg/m^3^ (cardiovascular mortality) [16]. Related to the *RR* for the short impact of PM_10_, WHO [17] recommends a value of 1.006 (95% CI 1.004–1.008) for all causes of mortality and all ages and a value of 1.009 (1.005–1.013) for cardiovascular disease. The Aphekom project [18] used for cardiovascular disease indicates a value of 1.006 (95% CI 1.004–1.008). The objective of this paper is to investigate the health risk assessment of PM_10_ and PM_2.5_ for the Central Region of Romania.

The suspended aerosol mass, in addition to PM_10_ and PM_2.5_, also contains new and emergent contaminants, such as nanoparticles and micro/nanoplastics. The nature and toxicity of suspended PMs, coupled with the presence of new and emergent contaminants, pose health impacts that can be very severe, especially for the very young, elderly, and people with immuno-compromised conditions. These health impacts include PM_2.5_- and PM_10_-induced airway inflammation, oxidative stress induced by polyaromatic hydrocarbons, covalent modifications of intracellular proteins/enzymes, the innate immune response, and inflammation by biologic compounds, adjuvant effects, suppression of normal defense mechanisms, suppression of oxygen transfer, and adverse impacts on the cardiovascular system and neurobehavior. Although a major source of PM is attributed to fossil fuel combustion and coal-burning stoves, the actual life cycle of suspended particulate matter, including new and emergent contaminants such as micro/nanoparticles [19] and micro/nanoplastics, is not well understood. The regional study conducted here can lead to mapping the health effects on a larger scale, and an integrated database may serve as the basis for expanded investigations into global health impacts due to PM and other suspended pollutants. Hence, this investigation lays the groundwork for developing some policies and guidelines regarding emission, exposure, and the use of associated personal protection equipment.

## 2. Study Area and Data Sets

**Figure 1:**
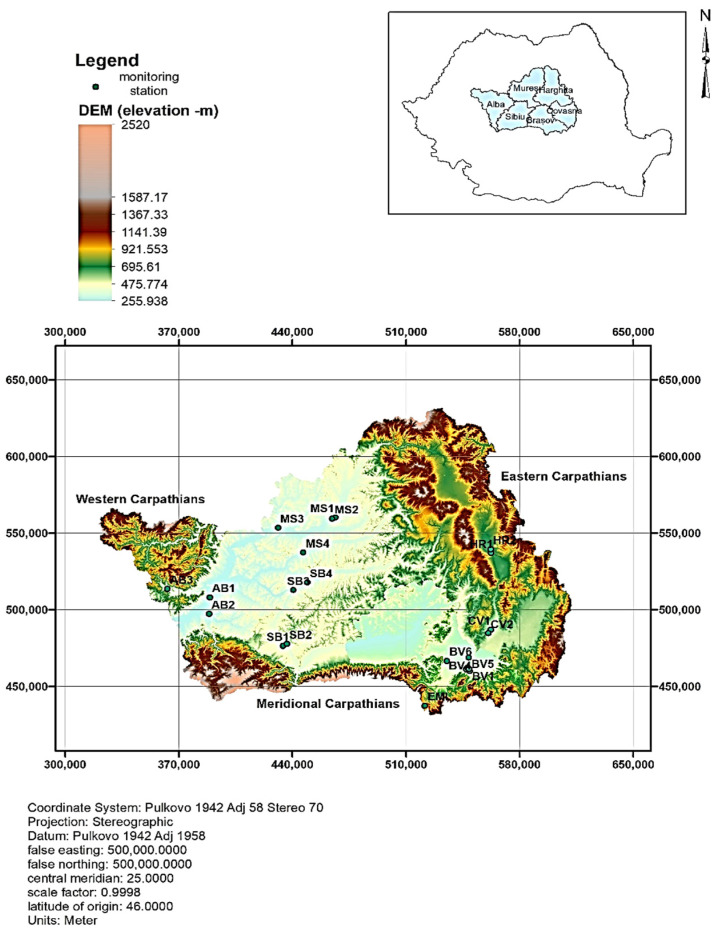
Elevation map of the Central Region of Romania and the location of monitoring stations.

The Central Region of Romania covers an area of 34,100 km^2^ and is located in the Carpathian Mountains’ inner arch (Figure 1). Parts of the three branches of the Carpathian Mountains, along with the hilly and depressed areas of the Transilvania Plateau form the relief, as shown in Figure 1. The hydrographic network is based on the Mures and Olt Rivers’ tributaries. Natural and anthropic lakes, such as Balea (glacier), St. Ana (volcanic), and hypersaline lakes, complete the hydrography of the Central Region. The climate is temperate continental and varies according to altitude. From an administrative point of view, the Central Region consists of Brasov, Sibiu, Alba, Mures, Harghita, and Covasna counties (Figure 1). On average, the population is estimated at approx. 2.6 million inhabitants. The region’s natural resources include natural gas, materials for construction (basalt, travertine marble), nonferrous metal, and numerous mineral springs. The vegetation consists of steppe; forests occupy about one-third of the territory (1185.1 thousand ha.). The economy has developed according to the resources of this area, such as industry (~32%), with the rest being allocated to agriculture, services, and tourism.

In this study, we use two types of datasets, grouped by independent and dependent variables. The independent variables include PM_10_ and PM_2.5_ as pollutants; the concentrations of these pollutants are obtained through the national air quality monitoring network [20] and correspond to daily data recorded between January 2012 and December 2021. The dependent variables include populations (ranging from age 30 to 85 years or more) and health data. Total mortality/morbidity and cardiovascular mortality data were collected from the National Institute of Statistics (NIS) over the same period (2012–2021) and within the same population age ranges. All data sets are available in the public domain without any attribution, and they do not contain any patient contact information and/or IDs.

The analysis of the status of principal air quality data and its correlation with the most affected area is based on the Romanian Network for Air Quality Monitoring, called Reţeaua Națională de Monitorizare automata a Calităţii Aerului (RNMCA), comprising 148 stations that survey air quality by measuring the concentration of principal pollutants. In the Central Region, there exist 22 stations distributed as follows: Brasov (BV)-seven, Sibiu (SB)-four, Alba Iulia (AB)-three, Mures (MS)-four, Harghita (HR)-two, Covasna (CV)-two, as presented in Figure 1 and Table 1. Unfortunately, the PM_2.5_ pollutant is not measured at every station. In Table 1, we introduced “yes” (Y) or “no” (N) to indicate whether the monitoring station measures this specific pollutant or not. It should be noted that at the HR2 station, PM_10_ is not measured. The reference method for sampling and measuring PM fractions is the one provided in the standard SR EN 12341 (available at https://magazin.asro.ro/ro/standard/229855, accessed on 12 September 2023), viz., ambient air. This standardized method involves gravimetric measurement for the determination of the mass fraction of PM_10_ or PM_2.5_ in suspended particles (in Romanian). All values are provided in µg/m^3^.

It is important to mention the accepted limits according to Romanian legislation (Law #104, which translates to national legislation Directive 2000/60/EC, Water Framework Directive [21] and 2004/107/CE [22] provisions): the standard daily limit for PM_10_ is 50 µg/m^3^, and 40 µg/m^3^ is the annual limit; the standard annual limit for PM_2.5_ is 25 µg/m^3^, and the target until (and after) 2020 is 20 µg/m^3^. The standard daily limit for PM_2.5_ is not regulated. The World Health Organization (WHO) has established guidelines on outdoor (ambient) air pollution levels. These guidelines, initially established in 2005 and updated in 2021, offer the recommended limits for airborne particulate matter, as shown in Table 2.

## 3. Methods and Methodologies

Based on the data sets, the methodologies proposed for this study include the following: (i) analysis of the status of PM_2.5_ and PM_10_ pollutants, (ii) analysis of total mortality and cardiovascular mortality, and (iii) health risk assessment. The analysis of PM_10_ and PM_2.5_ data consists of the following: (i) evaluating yearly and monthly PM_10_ and PM_2.5_ concentrations based on daily measurements; (ii) assessing descriptive statistics; (iii) assessing the number of days/year that exceed the limit value, as established by Romanian legislation and WHO requirements; and (iv) assessing the correlation between PM_10_ and PM_2.5_. Descriptive statistics are conducted using a data analysis package available with MS Excel 365.

To analyze total mortality and cardiovascular mortality, two indicators are used: (i) the mortality rate and (ii) cardiovascular mortality. The two rates are calculated as the number of deaths by 100,000 inhabitants. Health risk assessment consists of two stages: (i) health risk assessment based on the Ostro methodology [14] for the short-term effect of PM_10_ or PM_2.5_, (ii) health impact assessment based on the Pascal methodology [11] implemented in the “Aphekom” project and available on their website [24] for the long-term effect of PM_2.5_. Detailed equations are available in the guidelines presented on the aforementioned website. According to Ostro et al. [14], the calculation of the number of deaths associated with exposure to PM_10_ (total non-external causes mortality) or/and to PM_2.5_ (total mortality) is carried out based on the following equation:

(1) $$N_{assigned}=AF\cdot N_{total}=\frac{\left(RR-1\right)}{RR}N_{total}$$

where *N*_assigned_ represents the number of deaths assigned to PM_10_ or PM_2.5_ pollutants, *AF* the attributable fraction, *N*_total_ is the number total of deaths, and *RR* is the relative risk. The relative risk (*RR*) is calculated with the following formula:

(2) $$RR=exp\left[\beta \left(X-X_{0}\right)\right]$$

where *X* and *X*_0_ represent the annual average of pollutant concentration and background concentration as baseline values, respectively (e.g., 10 µg/m^3^), and *β* represents the concentration–response coefficient or the CFR coefficient. For short-term exposure of PM_10_, Ostro et al. [14] proposed a value of 0.0008 for the CFR coefficient values. Anderson [17] estimated a *β* value of 0.00059 (+/−0.00019) for all ages and all-cause mortality, as recommended by WHO [3]. According to Pascal et al. (2013) [11], an impacted lifetime table is calculated using the following equation:

(3) Dnmimpacted=Dnm·e−β·∆x

where Dnmimpacted and *_n_D_m_* are the total number of deaths in the age group starting at age n and covering m years for the impacted and baseline life tables, respectively; for the present study, *m* = 10 is considered to cover a ten-year interval. Δ*x* is the decrease in the pollutant concentration in such a given scenario. In this study, a *β* value of 0.00059 (+/−0.00019) for PM_10_ and 0.000598 (+/−0.000299–0.000895) for PM_2.5_ is used. Two scenarios are used: (1) where PM_2.5_ yearly average is decreased to 5 µg/m^3^, and (2) where the PM_2.5_ yearly average is decreased to 10 µg/m^3^. Concerning long-term exposure to PM_2.5_, the coefficient used for total mortality is 0.005826, and for CVD, it is 0.011.

To obtain the spatial distribution of a parameter, a geographic information system (GIS) method was used. The Voronoi method (or Thiessen polygon) was used to assign a surface for each station. Following this, an individual weight was calculated and used to assign the number of total deaths to a polygon. The Thiessen polygon method assumes that the parameter of each point is the same as that of the adjacent station measurement. The investigation was conducted for the period 2011–2022. Concerning data series representing mortality by age group, the National Institute of Statistics (NIS) did not calculate those parameters for 2011.

## 4. Results and Discussion

In Table 2, we have presented some statistical information about the pollutants measured at the 22 stations and the number of days/years that are over the limits recommended by Romanian legislation and the World Health Organization [21,22,23]. As can be seen, additional data are needed for the time series data sets in Table 1 and Table 3. During the study period, the multi-annual average concentration of PM_10_ varied between 9.41 µg/m^3^ (at Fundata station) and 30.95 µg/m^3^ (at Alba Iulia AB2). Generally, annual values of PM_10_ are not over the limit of 40 µg/m^3^, as required by Romanian legislation and the European Directive (with two exceptions for Brasov, viz., BV3 and BV5). These findings are in line with the results obtained by other authors for Romania [25,26]. Both the daily limit value and annual limit value recommended by WHO were also used as benchmarks in this study (Table 2 and Table 3a). Annual values of PM_10_ exceeded the annual safety limits required by WHO. The number of days over the daily limit (50 µg/m^3^) varied between 24% (AB2) and 0.5% (EMI–Fundata–Brasov). The number of days over the daily limit required by WHO in 2021 (45 μg/m^3^) varied between 18% (AB2) and 1% at the Fundata station. Concerning PM_2.5_, the average value for the Central Region is 17.48 µg/m^3^, continuing to surpass the thresholds recommended by the World Health Organization (WHO), which are set at 10 μg/m^3^ and 5 μg/m^3^, respectively [3,23]. The annual value of this pollutant is not over the limit of 25 µg/m^3^ for Sibiu (SB1) but exceeds the limit for all the other stations. The annual limit of PM_2.5_ concentrations exceeded for all stations, and the excess percentages were in the range of 11–100% (Table 3b).

**Figure 2:**
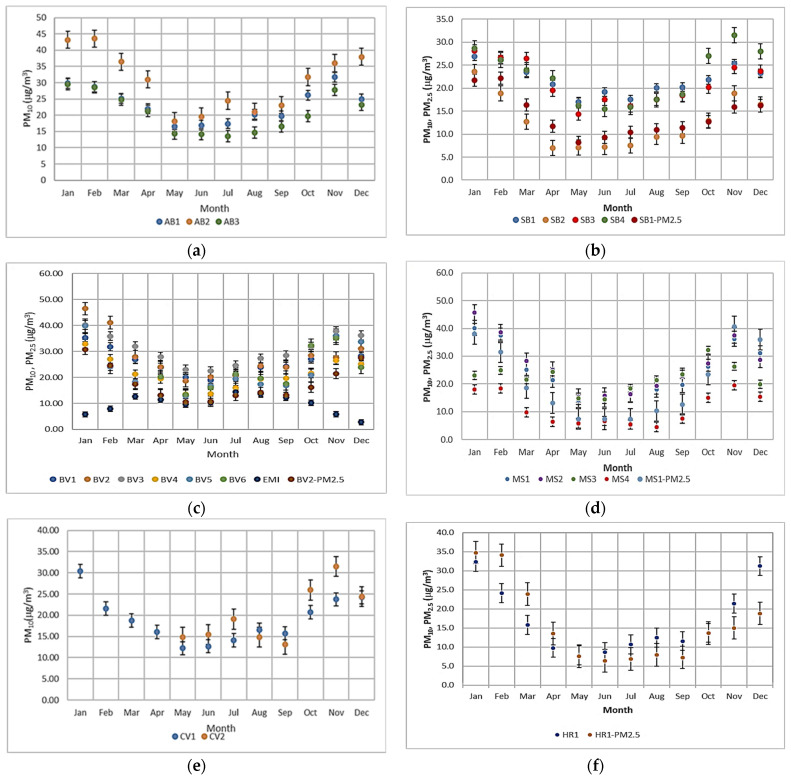
Evaluation of month-of-year mean concentrations of PM_10_ and PM_2.5_ over multiple years for (**a**) Alba, (**b**) Sibiu, (**c**) Brasov, (**d**) Mures, (**e**) Covasna, and (**f**) Harghita.

Investigating the multi-annual monthly values of PM_10_ and PM_2.5_, it was established that during the last spring period (May) and summer periods (June–August), the values of the two pollutants were lower than those observed in the rest of the year (Figure 2). These results are in agreement with the results obtained for Brasov by Maftei et al. [2] and for Cluj by Levei et al. [25]. In the autumn, winter, and earlier spring periods, the multi-annual monthly values are higher than the annual limits recommended by WHO in 2006 and 2021 [3,23]. Several studies indicate that variations in PM concentration throughout the seasons directly and adversely affect human health [27]. Only one exception exists in these datasets: the EMI station located in Fundata (Brasov County), where the variation of the PM_10_ pollutants shows a reverse trend. In January, February, November, and December, the multi-annual mean values are lower than the values registered during the rest of the year, when the multi-annual mean values are comparable with those in the rest of the monitoring stations. The influence of precipitation on PM_10_ was highlighted by Popescu LL et al. (2022) [28] through the daily measurements, while daily average PM_2.5_ concentrations were less influenced.

To estimate the relationship between PM_2.5_ (as a dependent variable) and PM_10_, regression analysis using the Excel data analysis package is employed. Considering the values of the R-squared determination coefficient, the results show that between 63% (MS1 and SB1) and 91% (BV2), the PM_2.5_ values fit the regression analysis model. The correlation coefficient of the linear relationship between the two variables is situated in the 0.79 and 0.95 range, which demonstrates a strong positive relationship. The F-significance value for all stations investigated is less than 0.05 (5%), which means that the null hypothesis is accepted, and the linear regression model fits the data well. Investigating the PM_2.5_/PM_10_ ratio, it is observed that this parameter varies within a narrow range (0.61 at SB1 station to 0.88 at MS1 station). The average ratio for the Central Region is 0.71, which is slightly over the value obtained by Bodor [12] for the same region (0.67) and in accordance with the value proposed by Pascal et al. (2013) in the Aphekom project [24].

The estimated population in the Central Region of Romania is approximately 2635 thousand inhabitants. The average population by county (Table 4) varies between 204,688 inhabitants (Covasna) and 551,685 inhabitants (Brasov). Generally, the population is decreasing (between −2.13% and −4.19%), with two exceptions: Brasov and Sibiu, where increases of 1.7 and 1.5%, respectively, were observed.

**Figure 3:**
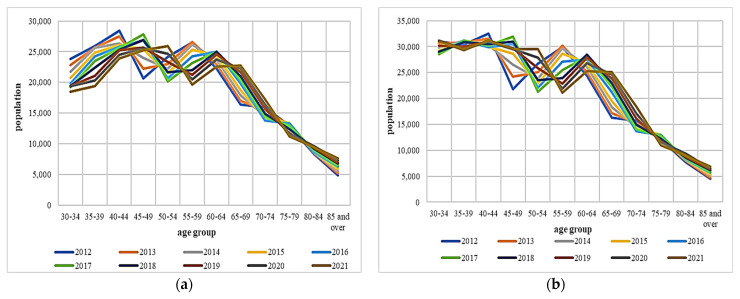

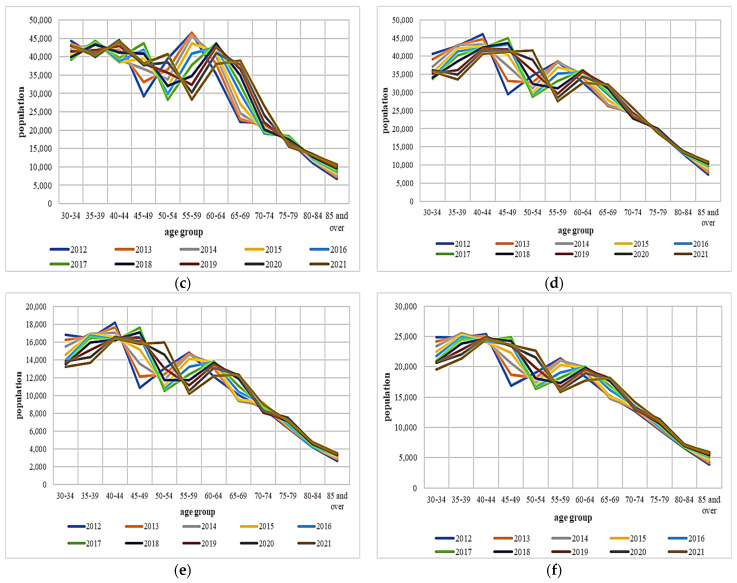
The population reported by age group for counties (**a**): Alba, (**b**) Sibiu, (**c**) Brasov, (**d**) Mures, (**e**) Covasna, and (**f**) Harghita.

The adult population by age group is presented in Figure 3. On average, more than 3% of the population around the Central Region was over the age of 80. The population aged 65 and above is between 13% (Sibiu) and 17% (Alba Iulia). Brasov has the highest population in the age group of 60–64 (6%). Moreover, the aging coefficient (its definition and explanation are beyond the scope of this article), physiologically, at the cellular level, is affected by the loss of specific regenerative and bioprotective mechanisms that occur over time in an organism due to exposure to PM. This coefficient varies between 19% (Sibiu) and 23% (Alba). Even while these percentages may seem negligible, the proportion of the elderly has increased significantly since 1960 (6.7%). The population aged under 30 is around 40%, with two exceptions: Covasna (20%) and Sibiu (24%). Moreover, Covasna has the lowest percentage in the age groups of 30–34, 35–39, and 40–45, which are ~7% each, respectively (Figure 3).

**Figure 4:**
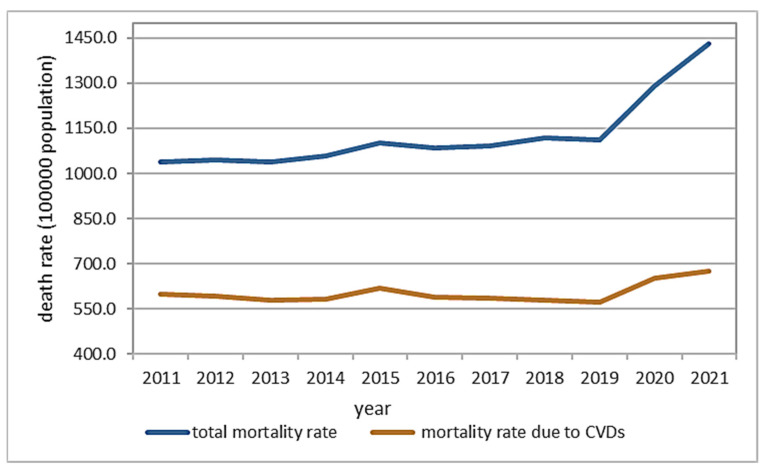
National mortality rate (2011–2021) (total mortality rate and rate due to CVDs).

**Figure 5:**
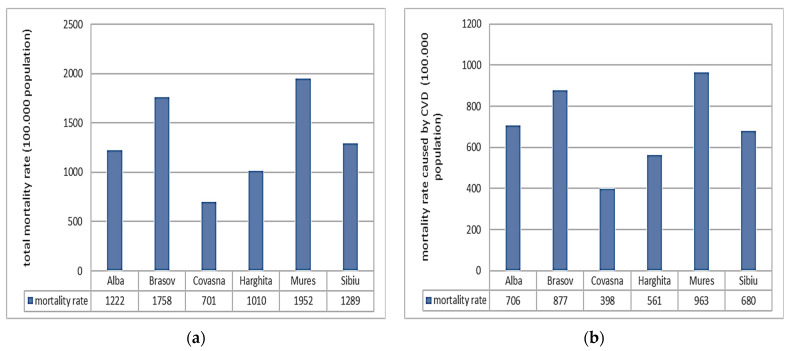
The total mortality rate (**a**) and mortality rate caused by CVD (**b**).

The national mortality rate is presented in Figure 4. For the study period (2011–2021), 53% of the total mortality was caused by cardiovascular diseases (CVD) [29]. A positive trend is observed in the total mortality rate (Figure 4) caused by fatalities registered in 2020 and 2021 (note: the time series data were reviewed by NIS—but are not final). The same behavior is seen for the mortality rate due to CVD (Figure 5). The pandemic’s indirect impact on the management of cardiovascular disease could partially be responsible for the higher-than-expected mortality toll associated with COVID-19 [30]. The total mortality rate by county is presented in Figure 5a. As can be seen, Mures County has the highest rate of total mortality (1925 per 100,000 inhabitants), followed by Brasov (1723 per 100,000 inhabitants). The lowest rate is registered in Covasna County. The mortality rate caused by CVD is presented in Figure 5b. As can be noticed, the highest mortality rate caused by CVD is registered in Mures County, while the lowest is in Covasna.

**Figure 6:**
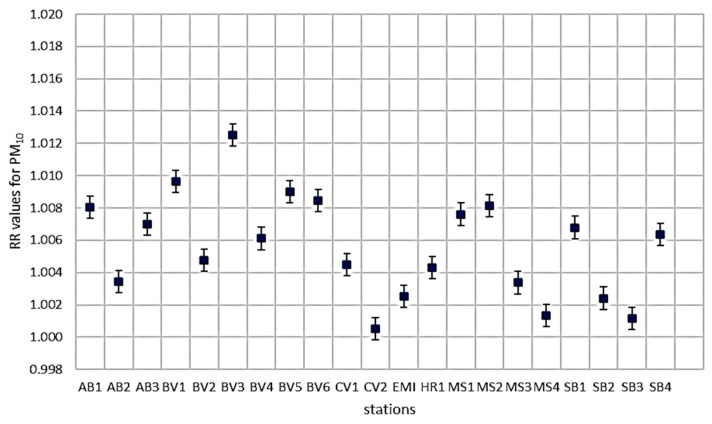
*RR* values for PM_10_.

**Figure 7:**
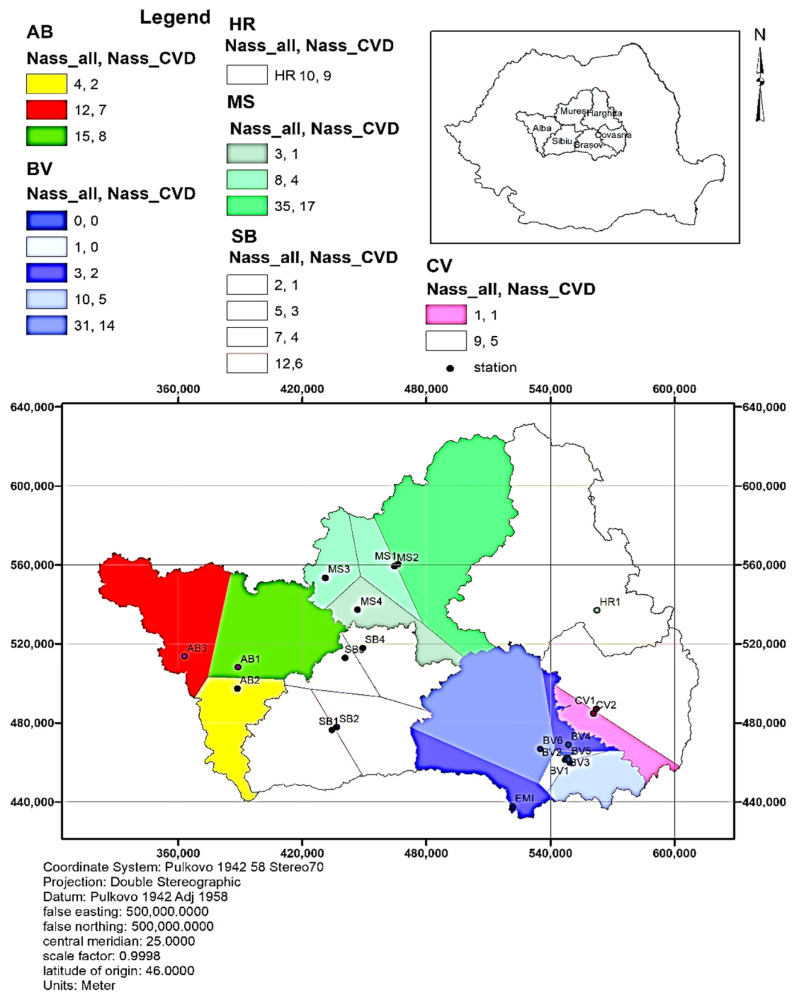
Spatial distribution of number of deaths attributed to all causes of mortality. (N_ass_ all) represents the number of deaths due to PM_10_ and (N_ass__CVD) represents the number of CVD-related deaths due to PM_2.5_.

The relative risk (RR) for PM_10_ and PM_2.5_ was estimated for all-cause mortality, and the results are presented in Figure 6 and Figure 7. Related to PM_10_, RR for HR2 is not calculated due to missing data.

Summary statistics related to PM_10_ *RR* values are presented in the following table (Table 5). The average value of *RR* is 1.006 (+/−0.0014), which is in agreement with the research literature, as mentioned in the Section 1. The minimum value is obtained for CV2 (1.001), which is situated in an urban (residential) area near Elisabeta Park. Research on the effect of urban greenery on air pollution conducted by Cohen P. (2014) demonstrates that the urban park could influence the PM_10_ and NOx concentrations [31].

In Brasov’s urban region, an average relative risk of 1.009 was observed in connection with PM_10_ and all-cause mortality. The highest recorded value was at BV3, situated near the train station—an area characterized by heavy road and rail traffic (1.013). The values obtained for urban areas in major cities (Sibiu, Târgu Mureș) are similar to those obtained in Brasov. Concerning PM_2.5_, there are only five stations that do not cover all the region. For a short-term analysis, the values of *RR* are similar due to *β* coefficient, which is very close to the coefficient used for PM_10_. For long-term exposure to PM_2.5_, the values of *RR* are shown in Table 6.

The spatial distribution of the number of deaths attributed to air pollution with PM_10_ (total non-external causes mortality)—N_assigned_ is presented in the following figure (Figure 7). The number of total deaths attributed to air pollution with PM_10_ varied from 53 (MS-Mures) to 10 (CV-Covasna) and the same parameter related to CVD varied from 26 to (MS-Mures) to 6 (CV-Covasna).

The number of deaths associated with exposure to PM_2.5_ (all causes of mortality) and PM_2.5_ (CVD mortality) for long-term analysis are presented in Table 7. The values represent the average for the period of study. As we mentioned in the methodology, to calculate the health impact assessment of PM_2.5_ for the long term, the methodology proposed in the “Aphekom” project was used. Within this method, PM_2.5_ is computed from PM_10_ using a correction factor of 0.7. In this case, we could use the incomplete time series data for the BV6 and HR2 stations. An example of such results, by this proposed method, is presented in Table 7.

**Figure 8:**
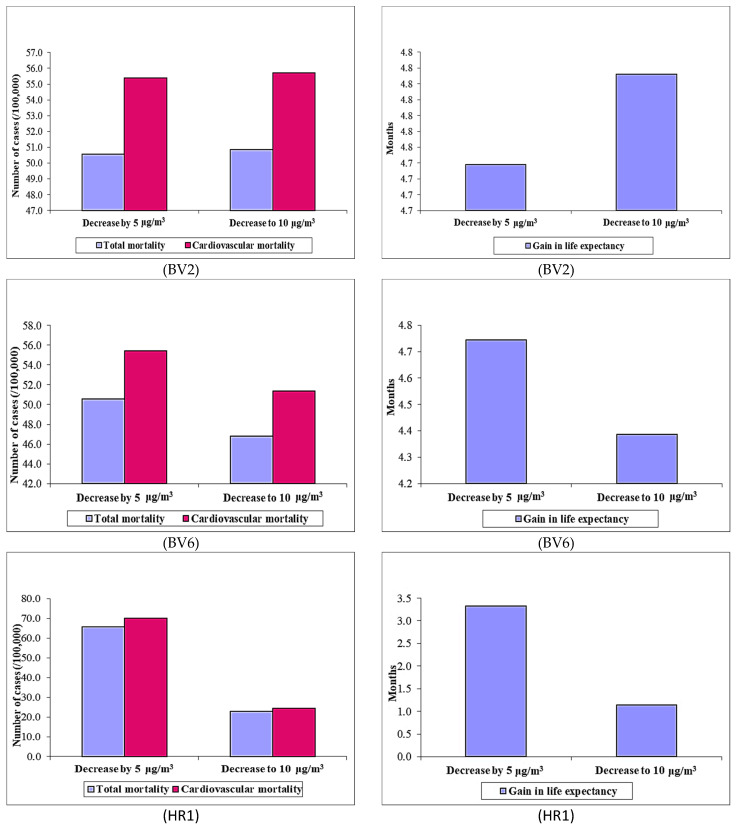

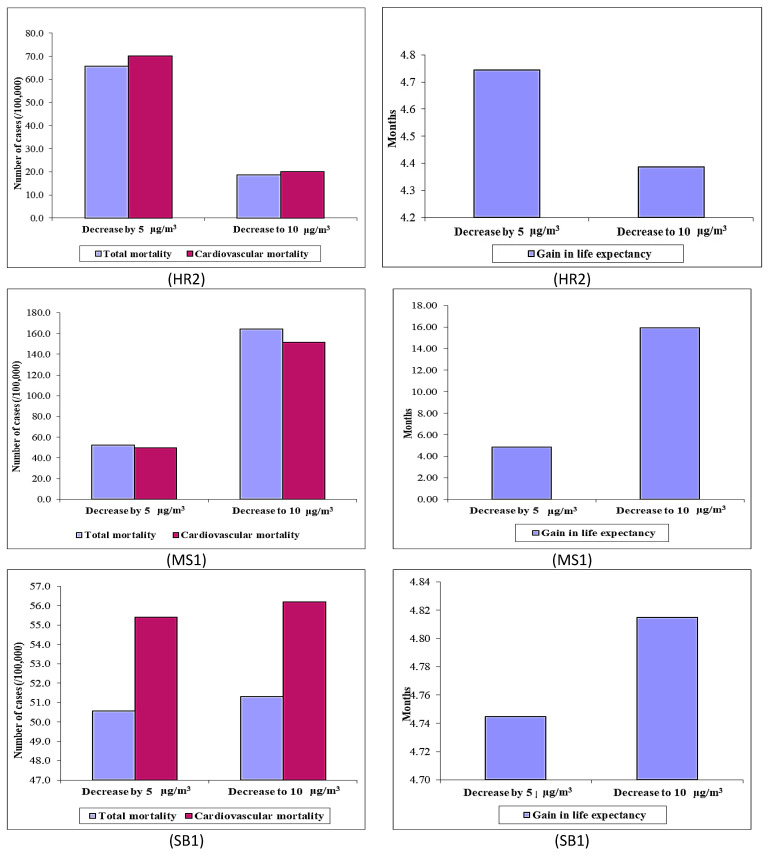
Potential benefits of reducing annual PM_2.5_ levels on total non-external mortality (**left**) and on total cardiovascular mortality (**right**).

Using the ratio PM_2.5_/PM_10_ of 0.7, the annual number of deaths avoided (for both all causes and CVD mortality) for the first scenario (viz., decrease by 5 μg/m^3^) remains the same for the period of this study. For the second scenario, it has been found that there is a decrease of 6 to 97.6%. The annual number of fatalities avoided due to the reduction of PM_2.5_ by 5 μg/m^3^ and to 10 μg/m^3^ (for both all causes and CVD mortality) is presented in Figure 8 for the study period. Decreasing air pollution levels (due to the reduction in PM_2.5_ by 5 μg/m^3^) to the updated WHO limits can save 161.5 lives (on average) in the case of total mortality (viz., 132.4—HR1 and 186.7—MS1) and an average of 147.5 (viz. 0.0—HR2 to 188.9—BV6) in the case of cardiovascular mortality. 

For the second scenario (with a decrease to 10 μg/m^3^), a total of 196.1 deaths are avoided in the case of total mortality, and approximately 191 deaths are avoided for cardiovascular mortality. The gain in life expectancy is on average 4.3 months for the Central Region in the first scenario, both for all causes and CVD mortality. In the second scenario, the gain in life expectancy is 5.3 for total mortality and 3.5 for cardiovascular mortality.

Health benefits that are related to an improvement of ambient air quality in the Central Region of Romania are similar to previous estimates obtained from different other studies conducted worldwide [25,28,32,33,34,35,36,37]. According to our study, a reduction in short-term exposure to PM_2.5_ by 5 μg/m^3^ results in an annual avoidance of non-external deaths ranging from 50.6 to 65.7 per 100,000 inhabitants. If cities situated in the Central Region of Romania could lower the mean of PM_2.5_ levels to 10 μg/m^3^, approximately 196 annual deaths (total non-external mortality) would be delayed, and the population would gain more than 5 months in life expectancy. Those values are in concordance with the results published on the ISGlobal Ranking of Cities website (https://isglobalranking.org**/**, accessed on 12 September 2023). Based on our investigations, if the cities in the Central Region of Romania managed to meet the WHO limits for PM_2.5_, approximately 198 deaths due to cardiovascular diseases could be avoided annually.

## 5. Conclusions, Limitations, and Future Directions

This article presents the health impact of an average of PM_10_ and PM_2.5_ levels above the average limits recommended by Romanian legislation and WHO in the Central Region of Romania. During this study period, the average of PM_10_ was observed to be 1.09 times higher than the annual acceptable limit of 20 μg/m^3^, which is 1.46 times higher than the annual acceptable limit of 15 μg/m^3^ (limits recommended by WHO 2006 and 2021, respectively). The maximum values of PM_10_, reaching 30.95 μg/m^3^, were registered in Alba Iulia County at the AB2 station, which is situated in Sebes town. This town is located near the intersection of two major highways (A0 and A1) and has a well-developed industrial complex nearby (especially in the wood industry). The second place is the BV3 station located in an urban city area (Brasov County) in proximity to the central railway station, with a significant amount of traffic nearby. The minimum value was registered at EMI-Brasov at 9.2 μg/m^3^; however, this station is a reference point for air quality assessment, being positioned at ~1350m elevation in a mountainous region of Brasov County. The multi-annual value of 10.21 μg/m^3^ is registered in Mures County at MS4. The location of this station is Tarnaveni town. The industry developed here is based on methane gas resources, albeit significantly diminished after the 1989 Romanian revolution. The 10 μg/m^3^ yearly limit of PM_2.5_ recommended by WHO (2006) [3] was exceeded by ~1.1 times at the SB1 station, located in a residential area in Sibiu city. The limit of 5 μg/m^3^ (WHO 2021) was exceeded by ~2.2 times at the same station and at the BV2 station, which is situated in a residential area in Brasov City. The monthly variation of PM_10_ and PM_2.5_ shows a strong seasonality in all six counties. The maximum level was registered in winter and autumn due to commercial and institutional activities, as well as household heating and transportation. It is instructive to note here that the Brasov area is a tourist destination due to the predominance of mountains. Several ski resorts have been developed here, which, along with the historical monuments, have led to the development of cultural tourism [25]. Despite tourism-related traffic, the minimum level was registered during the summer period.

Related to the calculated risk, two analyses were conducted. One refers to the short-term exposure of PM_10_/PM_2.5_, and the second to the long-term exposure effect of PM_2.5_. The higher calculated risk for PM_10_ risk was found in Brasov at three stations (BV3, BV1, BV5, and BV6). The first five stations mentioned here are located in Brasov city, while BV6 is located in Codlea town. Also, it is important to note that DN1—a national road—is an important source of pollution. This analysis provides the short-term calculated risk for PM_2.5_ (cardiovascular disease), and it is not significantly different from the PM_10_ risk, especially due to the CFR coefficient, which is practically the same. The higher calculated risk for PM_2.5_ for total mortality is obtained for MS1 and the two stations situated in Harghita County. The results show that exposure to these pollutants could cause an increase in both total mortality and cardiovascular mortality in the Central Region of Romania. The higher number of deaths assigned to PM_2.5_ is obtained in Mures—49 (on average), of which 25 are due to cardiovascular disease. As a result of long-term exposure to PM_2.5_, a higher number of deaths assigned to PM_2.5_ pollutants is obtained in Mures, ~616, of which 568 are due to cardiovascular disease. To conclude, Mures County is the most exposed county to PM_2.5_, followed by Brasov and Alba Iulia. The number of deaths assigned to PM_10_ pollutants varies from 10 in Covasna County to 56 in Mures County in terms of total mortality, from which 6 and 26, respectively, are assigned to cardiovascular mortality.

The results of this study offer the strongest argument currently available that prolonged exposure to airborne fine particulates that are typical of many urban areas is a significant risk factor for cardiovascular death. Related to health impact assessment, the present study shows that, by adopting the new WHO [3] limits, the potential benefits of reducing annual PM_2.5_ levels on total mortality are 196 (an average) in the Central Region of Romania. This would increase life expectancy by approximately 5.3 months. Related to cardiovascular mortality, the average number of deaths reduced is 190, which means an approximate 3.5-month increase in life expectancy. Hence, the results of this investigation indicate that there is a need to reduce the risk of various health concerns that could arise from exposure to particulate matter by introducing the new limits recommended by WHO in Romanian regulation.

Notwithstanding these observations in Central Romania, the study has several limitations, which include gaps in the data series, especially in the one related to PM_2.5_, which can lead to the establishment of an imprecise PM_2.5_/PM_10_ ratio. However, the results related to this ratio are slightly over the value established by Bodor et al. 2022 [12]; for this reason, we used 0.7 as the PM_2.5_/PM_10_ ratio. The small set of observations and/or lack of a complete dataset of PM_2.5_ measurement stations prevented the establishment of a realistic spatial distribution of this pollutant. In addition, there is a lack of studies on new-generation heating systems and initiatives due to green and economic policies, and several studies are incomplete. This is due to the inherent fact that the speed of technology far outpaces policies and regulations. Another limitation of the study is that since the data were analyzed as received from the stations, parametric confounding among variables could not be addressed due to the availability of limited data. To conduct a systematic statistical analysis, more datasets would be required. Thus, we believe that having a larger sensor density is crucial. Concerning the influence of climate parameters (precipitation, wind, temperature, etc.) on PM, we consider that modeling the dispersion of pollutants is one aspect that can add value to the studies.

As a future expansion of the scope of this study, it is important to consider that suspended aerosols now contain micro/nanoparticle and micro/nanoplastics, since such materials are used due to their unique properties, enabling a broad range of possible applications, including cosmetic, pharmaceutical, and medical utilization. Although a discussion concerning their emission mechanisms is beyond the scope of this article, these materials are emitted into the aquatic environment and air. The toxicological impacts of these new and emerging contaminants are largely unknown in terms of their health effects. Some preliminary studies, especially in aquatic environments [38,39,40], may serve as references to airborne contamination and its adverse health impacts. However, the overall study of the health impacts of PM_2.5_ and PM_10_, especially in the context of new and emerging contaminants, needs careful and extensive investigation. Furthermore, it will be very beneficial to quantify these data in terms of disability-adjusted life year (DALY)—as a measure of overall disease burden due to PM and new and emergent contaminants. For the aquatic environment, DALY indicators are estimated for such contaminants through the Universal Water Quality Index (UWQI) [41] for new and emergent contaminants.

## Figures and Tables

**Table 1:** Measurements of PM_2.5_ and PM_10_, along with monitoring station details such as type, location, and elevation.

No.	County/Station Indicative	Village	Type	Elevation (m)	PM_2.5_ (Y/N)
	**Alba**				
1	AB1	Alba Iulia	urban	246	N
2	AB2	Sebes	urban	256	N
3	AB3	Zlatna	urban	450	N
	**Sibiu**				
4	SB1	Sibiu	urban	430	Y
5	SB2	Sibiu	industrial	402	N
6	SB3	Copsa Mica	industrial	285	N
7	SB4	Medias	industrial	320	N
	**Brasov**				
8	BV1	Brasov	urban	593	N
9	BV2	Brasov	urban	593	Y
10	BV3	Brasov	urban	593	N
11	BV4	Sanpetru	suburban	560	N
12	BV5	Brasov	industrial	593	N
13	BV6	Codlea	urban	567	Y (Since 2022)
14	EMI	Fundata	suburban	1350	Y (Since 2022)
	**Mures**				
15	MS1	Targu Mures	urban	329	Y
16	MS2	Targu Mures	suburban	304	N
17	MS3	Ludus	suburban	270	N
18	MS4	Tarnaveni	suburban	284	N
	**Covasna**				
19	CV1	Sf Gheorghe	rural	522	N
20	CV2	Sf. Gheorghe	urban	563	N
	**Harghita**				
21	HR1	Miercurea Ciuc	industrial	710	Y (Since 2017)
22	HR2	Miercurea Ciuc	urban	689	Y (Since 2019)

**Table 2:** Safety limits for PM according to different air quality standards.

		PM_2.5_ μg/m^3^	PM_10_ μg/m^3^
Romanian legislation and EU standards	annual average	25 (20 until (and after) 2020)	40
	24 h average	not regulated	50
WHO limits [3] 2005	annual average	10	20
	24 h average	25	50
WHO limits [23] 2021	annual average	5	15
	24 h average	15	45

**Table 3:** Descriptive statistic on the annual values of PM_10_ data sets; no. of days over safety limits (see Table 2).

(a)																																						
			PM_10_ (2011–2021)—Statistics																																			
No. crt.	Station Indicative		No. obsv.		Min. μg/m^3^		Max. μg/m^3^		Mean μg/m^3^		StD. μg/m^3^		5th μg/m^3^		95th μg/m^3^		No. of Days over Limit of 50 μg/m^3^				No. of Days over Limit of 45 μg/m^3^				No. of Year over Limit of 40 μg/m^3^					No. of Year over Limit of 20 μg/m^3^					No. of Year over Limit of 15 μg/m^3^			
																	no		%		no		%		no		%		no			%		no			%	
			1		2		3		4		5		6		7		8		9		10		11		12				13			14		15			16	
**Alba**																																						
1	AB1		3769		0.01		104.84		23.36		13.73		8.18		49.42		110		3%		361		10%		0		0		9			82%		11			100%	
2	AB2		982		2.88		120.41		30.95		17.80		11.30		66.86		107		11%		179		18%		0		0		3			100%		3			100%	
3	AB3		3601		0.38		74.03		20.92		12.24		7.45		47.87		51		1%		241		7%		0		0		6			55%		11			100%	
**Sibiu**																																						
4	SB1		3206		0.73		109		21.57		12.42		7.27		45.42		98		3.06%		171		5%		0		0		6			60.00%		10			100.00%	
5	SB2		2813		0.27		102.97		12.75		11.69		1.58		35.56		49		1.74%		75		3%		0		0		2			18.18%		3			27.27%	
6	SB3		3051		0.24		112.64		20.87		13.73		4.72		47.24		104		3.41%		214		7%		0		0		6			54.55%		8			72.73%	
7	SB4		2845		1.27		125.36		22.74		15.14		5.45		49.86		142		4.99%		272		10%		0		0		7			70.00%		9			90.00%	
**Brasov**																																						
8	BV1		3557		1.82		179.23		26.09		16.87		7.63		55.81		252		7.08%		369		10%		0				11			100.00%		11			100.00%	
9	BV2		1649		2.36		255.93		27.41		20.83		8.18		57.40		107		6.49%		181		11%		0				5			100.00%		5			100.00%	
10	BV3		3710		1.27		216.48		30.81		19.37		10.22		64.14		368		9.92%		589		16%		1		0.02		11			100.00%		11			100.00%	
11	BV4		3151		0.36		200.95		21.16		17.48		4.54		49.33		153		4.86%		205		7%		0				7			70.00%		10			100.00%	
12	BV5		1167		0.43		272.09		22.80		22.65		5.63		56.21		74		6.34%		91		8%		2		0.17		6			75.00%		8			100.00%	
13	BV6		249		5.09		80.36		22.06		13.01		7.73		46.68		11		4.42%		16		6%		0				1			100.00%		1			100.00%	
14	EMI		726		0.73		66.86		9.48		8.00		1.45		23.89		4		0.55%		5		1%		0				0			0.00%		0			0.00%	
**Mures**																																						
15	MS1		2126		0.89		154.32		24.10		17.80		6.22		58.44		170		8.00%		231		11%		0				8			72.73%		9			81.82%	
16	MS2		2438		0.52		263.71		25.19		18.98		5.79		60.47		210		8.61%		283		12%		0				8			72.73%		9			81.82%	
17	MS3		881		2.05		110.36		22.43		13.21		7.26		48.39		40		4.54%		54		6%		0				3			75.00%		4			100.00%	
18	MS4		2133		0.04		95.39		10.59		11.26		1.60		32.29		41		1.92%		57		3%		0				0			0.00%		3			33.33%	
**Covasna**																																						
19	CV1		2484		0.09		141.16		18.93		13.19		4.62		43.67		88		3.54%		118		5%		0				5			45.45%		8			72.73%	
20	CV2		204		1.63		75.25		21.85		19.7		5.71		41.04		6		2.94%		8		4%		0				0			0.00%		1			100.00%	
**Harghita**																																						
21	HR1		3225		0.18		221.6		16.94		19.05		2.36		51.02		164		5%		197		6%		0				2			18%		8			73%	
22	HR2		-		-		-		-		-		-		-		-		-		-		-		-				-			-		-			-	
**(b)**																																						
**Station Indicative**		**PM_2.5_ (2011–2021)—Statistics**																																				
		**No.** **obsv.**		**Min** **μg/m^3^**		**Max** **μg/m^3^**		**Mean** **μg/m^3^**		**StD.** **μg/m^3^**		**5th** **μg/m^3^**		**95th** **μg/m^3^**		**No. of Year over Limit of 25 μg/m^3^**				**No. of Year over Limit of 20 μg/m^3^**				**No. of Year over Limit of 10 μg/m^3^**		**No. of Year over Limit of 5 μg/m^3^**					**No. of Days over Limit of 25 μg/m^3^**					**No. of Days over Limit of 15 μg/m^3^**		
																**no**		**%**		**no**		**%**		**no**	**%**	**no**		**%**			**no**		**%**			**no**		**%**
		**1**		**2**		**3**		**4**		**5**		**6**		**7**		**8**				**9**				**10**		**11**					**12**					**13**		**14**
**Sibiu**																																						
SB1		2347.00		0.53		78.40		13.85		9.86		3.45		34.52		0		0%		1		11%		7	78%	9		100%			282		12%			791		34%
**Brasov**																																						
BV2		3283		1.09		198.31		17.36		14.67		4.9		41		1		9%		3		27%		11	100%	11		100%			564		17%			1521		46%
**Mures**																																						
MS1		1606		0.71		161.9		21.22		21.72		4		66.3		2		29%		3		43%		7	100%	7		100%			407		0.25			726		0.5
**Harghita**																																						
HR1		697		0.18		154.99		15.68		19.79		2.3		52		2		40%		2		40%		4	80%	5		100%			99		14%			200		29%
HR2		194		2.17		78.8		19.3		14.51		4.9		52.1		1		100%		1		100%		1	100%	1		100%			43		22%			90		46%

**Table 4:** Average population for Central Region counties (2011–2021).

	Central Region	Alba	Brasov	Covasna	Harghita	Mures	Sibiu
average	2,635,986	380,571	632,764	228,459	333,280	595,829	465,083

**Table 5:** Summary statistics of *RR* values related to PM_10_ for all monitoring stations.

Average	1.006	Maximum	1.0125
Standard Error	0.0007	Count	21
Median	1.006	CI (95.0%)	0.0014
Standard Deviation	0.00317	Upper Limit	1.0074
Minimum	1.00052	Lower Limit	1.0042

**Table 6:** *RR* values for the five stations investigated.

Station	*RR*		N_assigned_	N_assigned_ CVD
	All Causes	CVD		
**BV2**	1.044	1.088	272	259
**HR1 and HR2**	1.054	1.127	312	100
**MS1**	1.072	1.148	616	568
**SB1**	1.084	1.171	138	130

**Table 7:** Potential benefits of reducing annual PM_2.5_ levels on total non-external mortality and total cardiovascular mortality for Brasov.

Station	Scenarios	Total Non-External Mortality			Total Cardiovascular Mortality	
		Annual Number of Deaths Avoided	Annual Number of Deaths Avoided per	Gain in Life Expectancy (Months)	Annual Number of Deaths Avoided	Annual Number of Deaths Avoided per
			100,000			100,000
BV2	Decrease by 5 μg/m^3^	172.4	50.6	4.74	188.9	55.4
	Decrease to 10 μg/m^3^	173.4	50.9	4.77	190.0	55.7

## Data Availability

Most of the data are contained in the article. Any additional data, including links to publicly archived datasets analyzed or generated during the study, can be requested from the corresponding author, upon reasonable request.
